# Stimulus Requirements for Face Perception: An Analysis Based on “Totem Poles”

**DOI:** 10.3389/fpsyg.2013.00018

**Published:** 2013-02-12

**Authors:** Carrie L. Paras, Michael A. Webster

**Affiliations:** ^1^Department of Psychology, University of NevadaReno, NV, USA

**Keywords:** face perception, face detection, configural coding, facial features, symmetry, inversion effects, noise

## Abstract

The stimulus requirements for perceiving a face are not well defined but are presumably simple, for vivid faces can often by seen in random or natural images such as cloud or rock formations. To characterize these requirements, we measured where observers reported the impression of faces in images defined by symmetric 1/f noise. This allowed us to examine the prominence and properties of different features and their necessary configurations. In these stimuli many faces can be perceived along the vertical midline, and appear stacked at multiple scales, reminiscent of “totem poles.” In addition to symmetry, the faces in noise are invariably upright and thus reveal the inversion effects that are thought to be a defining property of configural face processing. To a large extent, seeing a face required seeing eyes, and these were largely restricted to dark regions in the images. Other features were more subordinate and showed relatively little bias in polarity. Moreover, the prominence of eyes depended primarily on their luminance contrast and showed little influence of chromatic contrast. Notably, most faces were rated as clearly defined with highly distinctive attributes, suggesting that once an image area is coded as a face it is perceptually completed consistent with this interpretation. This suggests that the requisite trigger features are sufficient to holistically “capture” the surrounding noise structure to form the facial representation. Yet despite these well articulated percepts, we show in further experiments that while a pair of dark spots added to noise images appears face-like, these impressions fail to elicit other signatures of face processing, and in particular, fail to elicit an N170 or fixation patterns typical for images of actual faces. These results suggest that very simple stimulus configurations are sufficient to invoke many aspects of holistic and configural face perception while nevertheless failing to fully engage the neural machinery of face coding, implying that that different signatures of face processing may have different stimulus requirements.

## Introduction

The ability to reliably identify individual faces and classify their attributes represents a pinnacle of performance in human visual perception, for the physical cues that distinguish faces are subtle, while the stimulus variations that result from changes in lighting or viewpoint or in the face itself (e.g., a change in expression) are pronounced. This capacity is thought to depend in part on configural processing, in which faces are represented by the spatial relationships between the elements of the face rather than simply a piecemeal analysis of the independent parts (Carey and Diamond, 1977; Sergent, 1984; Bartlett and Searcy, 1993). Classic sources of evidence for configural processing includes inversion effects, in which faces become less recognizable when turned upside down (Yin, 1969), and interaction effects in which changes to one part of the face alter the perception and recognition of other parts (Young et al., 1987; Tanaka and Farah, 1993). Such results suggest that in upright faces, information like identity is encoded holistically, yet the actual dimensions the visual system samples to extract this information remain elusive.

Configural processing has also been used to refer to the detection of a stimulus as a face, for this detection presumably involves recognizing the presence of requisite elements in particular spatial relationships (e.g., two eyes, a mouth, and a nose; Maurer et al., 2002). Here again the stimulus configurations that support face detection are not well defined. However, it is likely that these configurations are very simple, for it is often possible to experience vivid impressions of faces in random objects including natural formations (e.g., the man in the moon) or constructed objects (e.g., cars or faucets). Such illusory percepts are the most prominent example of pareidolia, in which random stimuli generate the perception of seemingly recognizable objects. The fact that faces can be seen so readily in random patterns may partly reflect their salience as a stimulus class, but must also arise because the stimulus configurations required to elicit them must be weak enough so that they can occur with high probability. Characterizing these requirements is of interest because it can point to the basic templates the visual system might use for the initial coding of a stimulus as a face.

In this study we explored when observers see faces in random patterns, by asking observers to detect faces in noise. The noise had a 1/f amplitude spectrum characteristic of natural images, and thus included salient but random spatial structure at many spatial scales (Field and Brady, 1997). In this filtered noise we found that faces can be seen but only infrequently and often appeared indistinct. We therefore added one non-random constraint to make the noise symmetric about the vertical midline. The images were thus similar to Rorschach patterns, though much richer in texture, and now gave rise to many and often dramatic impressions of faces. These occurred at multiple scales and with rare exceptions appeared stacked on top of each other along the axis of symmetry, analogous to the faces seen in “totem poles.” Our aim was to measure where in the images observers reported a face in order to assess the stimulus properties that were necessary to elicit a face percept.

A second aim was to assess what kinds of face percepts could be elicited by the noise. Faces are typically categorized along a number of attributes including species or individual identity, gender, expression, or age, and as noted this individuation is thought to depend on specialized and higher-order configural coding of the stimulus. We asked whether noise patterns necessarily engaged these processes by asking to what extent these higher-order and abstract attributes could be discerned in the noise. We also asked whether the noise showed the same diagnostic signs of configural processing that are ascribed to the perception of actual faces.

A final aim was to assess whether the impression of a stimulus as a face was sufficient to fully engage face coding mechanisms. The faces we imagine in clouds or rocks are rarely mistaken for actual faces, and as a result might lead to different perceptual processing. To test this, we examined to what extent the noise faces gave rise to patterns of responses that are typical of actual faces.

## Materials and Methods

### Subjects

Observers included 41 university students who participated for course credit and who were unaware of the specific aims of the study. Individual participants took part in different subsets of the experiments. All observers had normal color vision and normal or corrected visual acuity as assessed by standard screening tests and no known deficits in face recognition based on self-reports. Participation was with informed consent and followed protocols approved by the University’s Institutional Review Board.

### General stimuli and procedure

**Figure 1:**
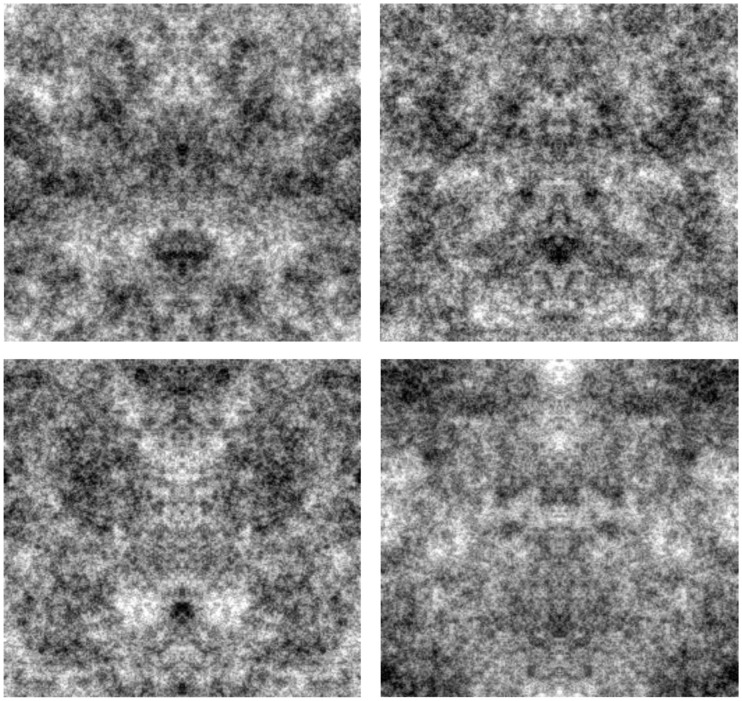
**Examples of the symmetric 1/f noise images**.

We explored a variety of images and tasks, with details specific to each experiment described below. Images in most cases consisted of 256 × 256 pixel 1/f symmetric Gaussian noise. Examples are illustrated in Figure 1. The images were initially generated by selecting 8-bit pixel values from random normal deviates but constrained to be symmetric about the vertical axis. These symmetric white noise images were then spatially filtered so that the amplitude spectrum varied as 1/f. After filtering all images were adjusted so that the mean value corresponded to 128 and the rms contrast equaled 35% of the mean. This scaling was near the limit to avoid significant pixel saturation, and only images with fewer than 0.5% truncated pixels were included as stimuli. A set of 100 images were created for each testing condition. The images were displayed at 512 by 512 pixels on an E540 Sony Trinitron monitor in an otherwise dark room, and were shown on a gray background of the same mean luminance. Observers were seated in front of the monitor and free-viewed the stimuli without time constraints while making their selections.

## Results

### Faces seen in noise

In the first set of experiments we examined the characteristics of faces perceived in the symmetric noise and the image properties that gave rise to them. These properties were estimated from >1000 faces identified across a large sample of the noise images judged by eight observers. The pattern of results was generally consistent across observers. Participants were instructed to scan the images to find regions that appeared to them to look like a face. For each face they found, they then used a series of drop-down menus to label its perceived characteristics. These included how distinct the face appeared and the perceived species. They were also asked to judge the perceived age, gender, ethnicity, and expression. Each judgment other than distinctiveness included a “not visible” option.

**Figure 2:**
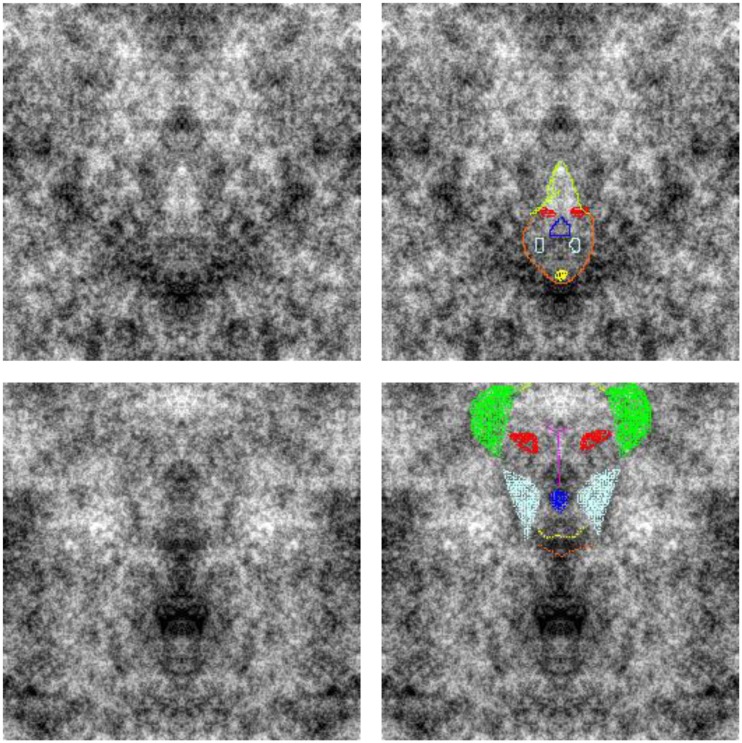
**Examples of the faces reported by observers**. After identifying and describing the face, observers drew on the image to indicate the perceived position and shape of features. Different features were coded with different colors (e.g., red for eyes, blue for nose, etc.) so that their dimensions could be analyzed afterward.

After completing this labeling, the observers next identified the visible features of the face from a menu that included eyes, nose, mouth, ears, eyebrows, and head outline. For each they used a graphics pad to point to the center of the relevant feature, whose coordinates were then recorded, and also used a drop-down menu to record the distinctiveness of the feature. Finally, they next used the graphics pad to outline or fill in the contours of each feature by drawing on the image. These drawings were stored by overwriting the pixel values in a copy of the image, using different colors for each chosen feature so that these could be distinguished for subsequent analysis. Examples of faces drawn by the observers (who varied widely in artistic talent) are illustrated in Figure 2. Once these steps were completed the original image was redisplayed so that observers could look for additional faces. Typically many faces were detected in a single image. When observers felt they could no longer find new ones, they advanced to the next image, and repeated the sequence of responses. Images were shown in random order both within and across subjects. The drawings were analyzed by fitting ellipses to each selected feature to estimate the position, orientation, and aspect ratio of the smallest ellipse that encompassed 95% of the chosen pixels. The luminance contrast of the feature was also estimated from the average pixel level within the best-fitting elliptical area encompassing the feature, relative to the average within surrounding annuli defined by an ellipse with 1.5 or 2 times the area.

#### Perceived facial characteristics

Participants often identified a multitude of faces within a single image. Moreover, the chosen faces were often rated as highly distinctive. In fact our procedure probably greatly underestimates the number of potential faces in the images since observers tended to restrict their choices to the most salient examples. These were often clearly identifiable as a specific, well defined individual. For example, Table 1 shows that the apparent species of the illusory faces was reported as unclear only 6% of the time, and that more than 20% appeared to be human. Moreover, the appearance of an identifiable expression was reported for 64% of the faces, while surprisingly an impression of age was reported almost without exception. In contrast, a gender was assigned much less frequently (20%), in part because many of the faces appeared to be a non-human species.

**Table 1:** **The percentage of faces described as appearing to have a particular attribute**.

Species		Expression		Age		Gender	
Mammal	44	Happy	26	Young	22	Male	17
Human	21	Angry	22	Medium	60	Female	3
Monster	16	Surprised	4	Old	17		
Other	6	Sad	9				
Unclear	6	Afraid	2				
Bird	3	Disgusted	<1				
Reptile	>1						
Fish	<1						
Insect	<1						
Total Responses	99%		64%		99%		20%

**Figure 3:**
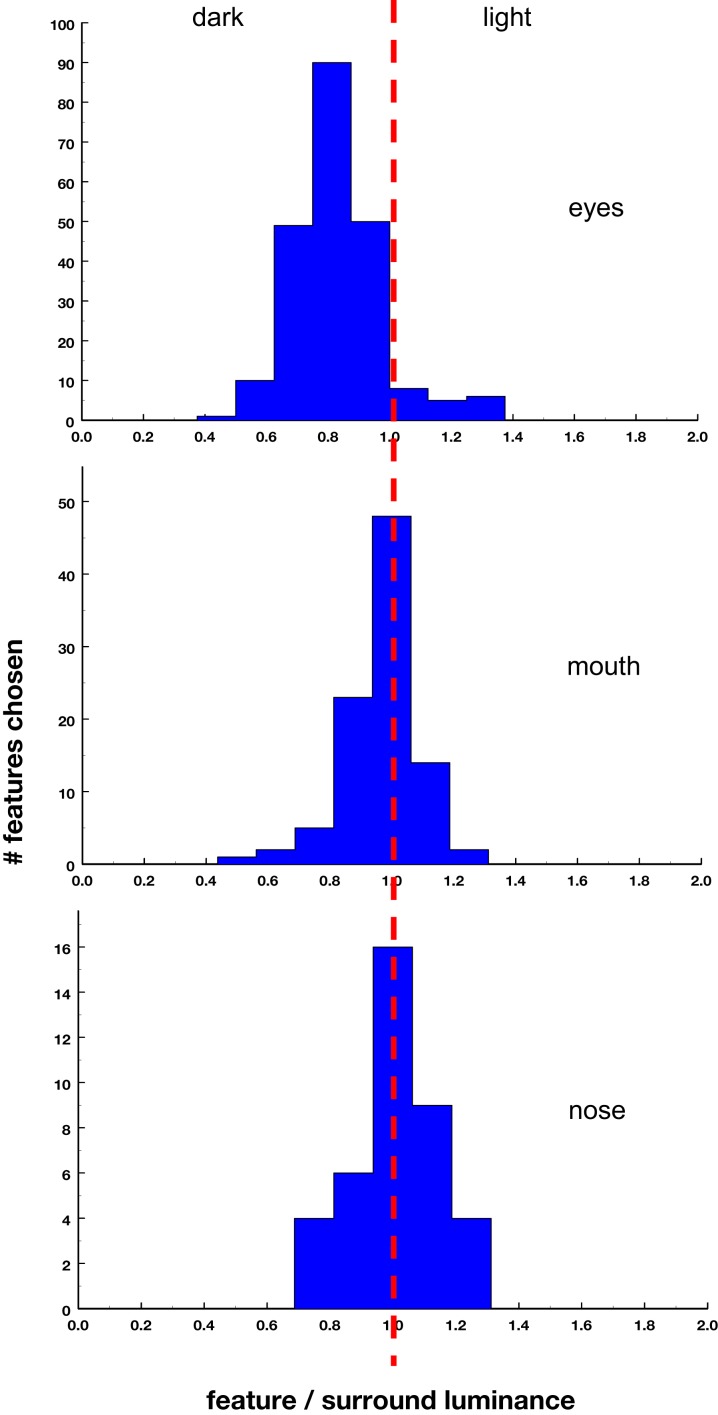
**Ratio of the average luminance (pixel gray level) of features relative to the average luminance of the surrounding annulus**.

Table 2 lists for eight observers the percentage of time that a particular feature in the face was reported. Eyes were by far the most prominent percept, averaging 95% and close to 100% for individual observers. These were followed by a nose or mouth, which were indentified much less consistently across observers (60–70% of the time). Finally, ears were identified by only two of the eight observers. Thus a pair of eyes, perhaps combined with either a nose or a mouth, appeared to be critical for eliciting a face percept. Moreover, the regions identified as eyes were strongly biased toward lower luminances (pixel values) than the local surround, or in other words to darker spots in the images (Figure 3). In contrast, there was not a significant bias in the luminance polarity of either the mouth or the nose. Again, this polarity was assessed by comparing the average pixel level within an elliptical region fit to the observer’s demarcation of the feature, relative to the average level measured within a surrounding annulus, and thus probably failed to capture the relevant levels in some faces, for example when the eyes were elicited by an outline or higher luminance variance rather than a uniform area. Nevertheless, the results suggest that in order to perceive a face, the image had to contain dark spots for the eyes, while the mouth or nose was defined by its spatial information but not by its contrast.

**Table 2:** **Detected features calculated as a percent of total faces reported for each subject**.

	Eyes (%)	Nose (%)	Mouth (%)	Ears (%)
S1	87	87	67	56
S2	97	82	73	0
S3	98	85	74	0
S4	99	26	30	0
S5	91	1	84	47
S6	95	92	73	0
S7	96	86	64	0
S8	94	90	58	0
Average	95	69	65	13

#### Perceived facial configurations

**Figure 4:**
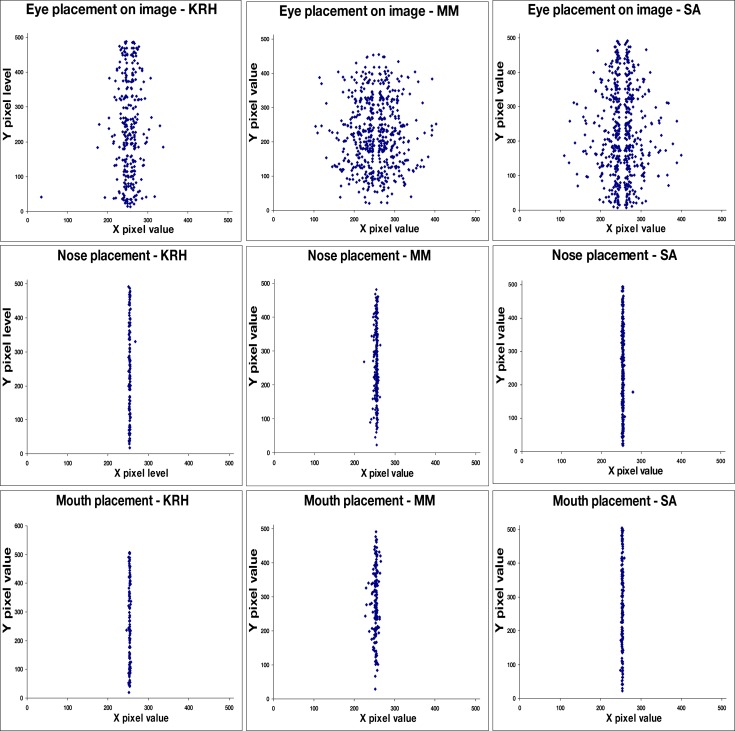
**Perceived locations (center pixel) of features in the images, illustrated for three observers**. Top: eye positions; middle: nose positions; bottom: mouth positions.

The faces identified in the images varied widely in spatial layout, configuration, and size showing that a large range of spatial configurations could appear as a face. Moreover, while we did not record perceived viewpoint, informal observations, and reports suggest that almost all faces were perceived as frontal views and thus not foreshortened, again suggesting that a wide range of configurations can elicit a face percept. For the symmetric noise images we tested, the faces reported were almost without exception frontal view images aligned to the axis of symmetry. This can be seen in Figure 4, which plots the location reported for the eyes, which were symmetrically positioned about the midline, in comparison to the nose and mouth, which were tightly confined to the midline. The paucity of percepts of face profiles is notable. One possibility is that profiles require stronger cues for the head outline than frontal faces, for which the internal face features may be sufficient. Within the noise we used there are not strong percepts of separate figure and ground regions which may therefore have worked against perceiving head outlines.

**Figure 5:**
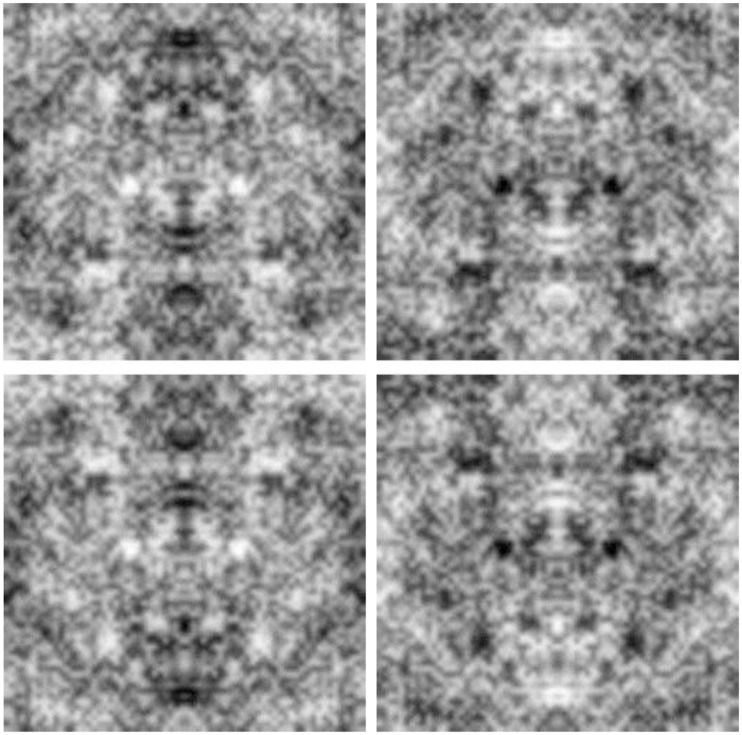
**An illustration of the effects of inverting the orientation or contrast polarity on the perceived faces in the images**. The top left image shows a sample from one of the original noise images. The top right shows the same image with the contrast inverted; the bottom left with the orientation flipped; and the bottom right with both the orientation and the contrast inverted.

Observers further invariably reported seeing upright faces, with the eyes above the nose and mouth. As a result, the faces perceived in the noise images replicate the hallmark inversion effects characterizing face perception. The effects of symmetry, orientation, and contrast are illustrated in Figure 5, which shows a magnified view of prominent faces from one of the noise images. The faces individuals report seeing in the four images are very different, though the images are all drawn from the same original noise sample and differ only because the orientation and/or contrast polarity has been inverted. In each case the specific faces seen are likely to be aligned to the axis of vertical symmetry and are likely to be upright and with dark eyes.

As noted, faces normally appeared as upright and frontal views. The horizontal distance between the eyes was, on average, five times greater than the vertical distance to the nose (Table 3), which is exaggerated in comparison to that of a human face (Robbins et al., 2007; Pallett et al., 2010). Since most of the faces chosen were not human (see Table 1), this might account for the fact that the average configuration was not of a human face. However this also suggests that faces do not need to conform to standard configurations or templates but rather can be detected for a wide range of stimuli. In this regard it is interesting to note that even human faces remain highly recognizable under global distortions of the image, such as vertically stretching the picture (Hole et al., 2002).

**Table 3:** **Ratio of the horizontal distance between eyes to the vertical distance from the eyes to the nose**.

	Average ratio	Standard deviation
S1	6.24	15.4
S2	5.03	6.79
S3	4.92	5.86
S4	7.65	9.90
S5	3.24	4.00
S6	5.10	7.12
S7	3.40	4.34
S8	4.18	5.96
Average	4.97	7.43

#### Polarity vs. symmetry

**Figure 6:**
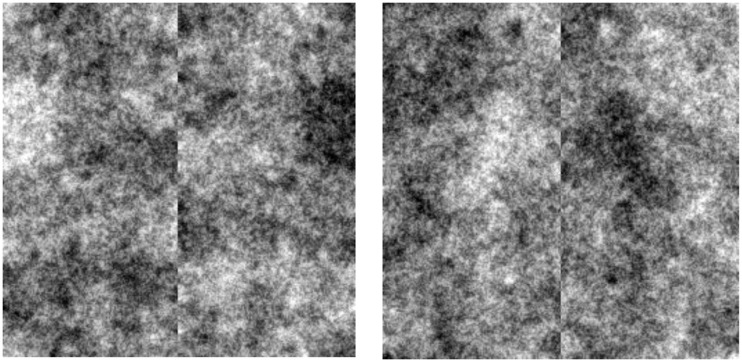
**Examples of spatially symmetric images with asymmetric contrast**.

**Figure 7:**
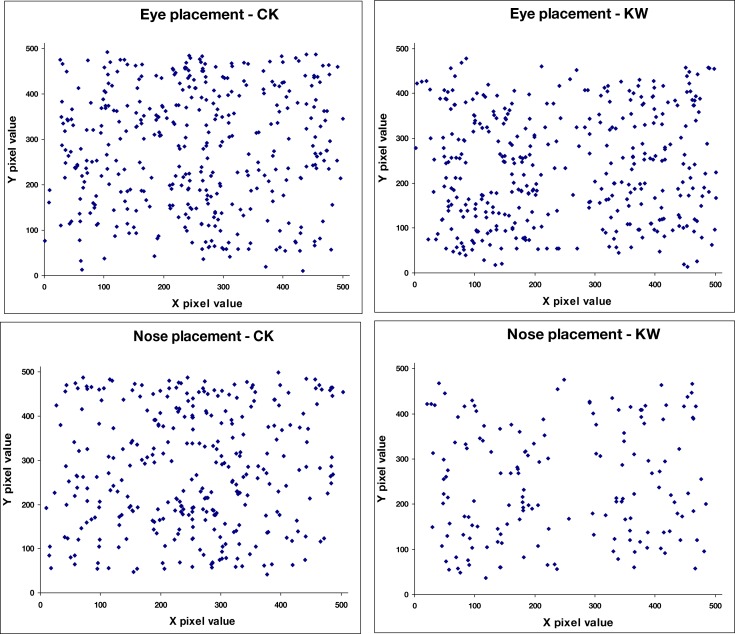
**Perceived location of eyes and nose in faces in the symmetric images with asymmetric contrast, illustrated for two observers**. Top: eye positions (center pixel); bottom: nose positions.

To assess the relative salience of these properties, we created a second set of images which were identical to the preceding symmetric noise except that the pixel level was inverted between the left and right sides (Figure 6). This allowed us to pit the cue of eye polarity and symmetry against each other. The same procedures were used to collect face percepts from four new observers. Under these conditions symmetry in fact ceased to be a factor and observers instead identified faces throughout the image, again driven by the dark polarity of selected eye regions (Figure 7).

#### Luminance vs. color

**Figure 8:**
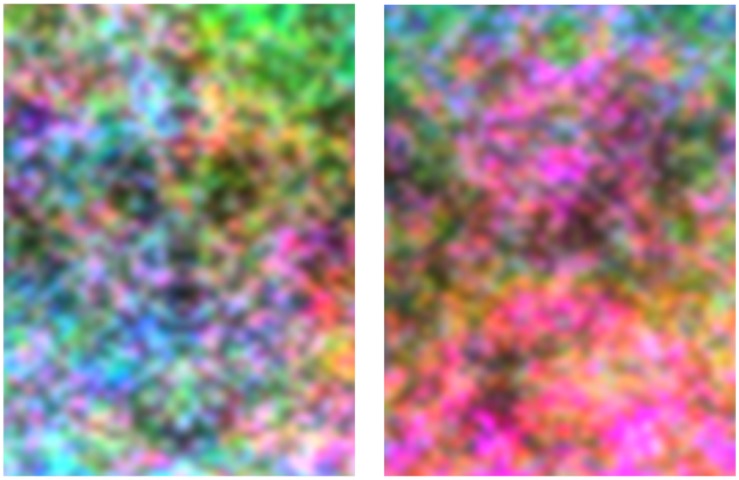
**An example of images with symmetric luminance contrast and random chromatic contrast (left), or symmetric color and random luminance (right)**.

**Figure 9:**
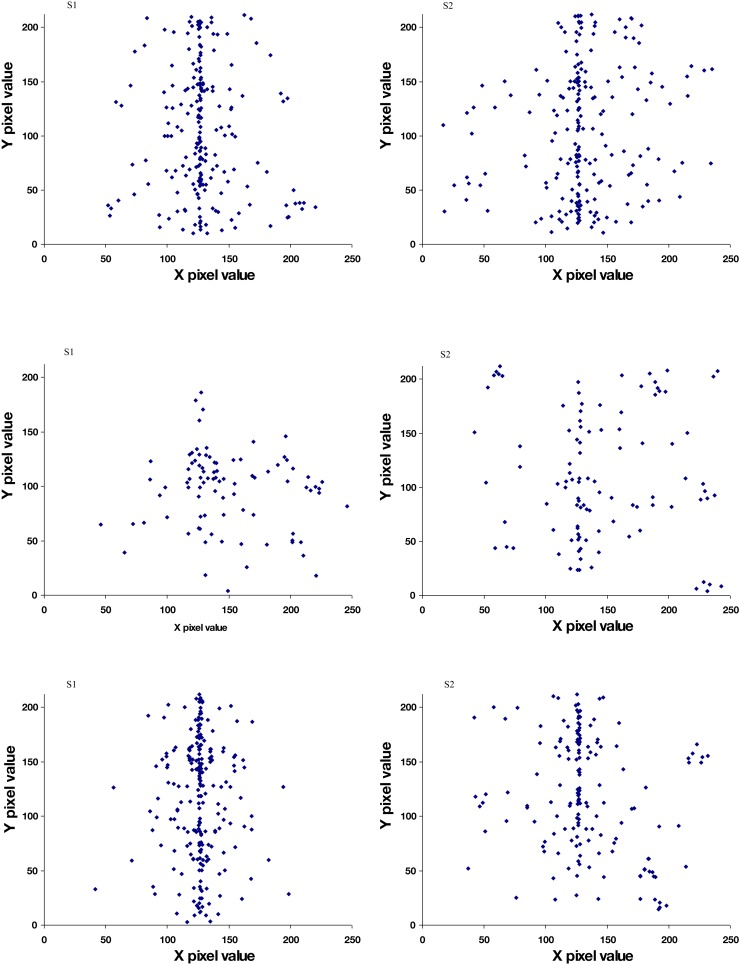
**Perceived location of eyes chosen by two observers in images with symmetric luminance variations but random color (top); symmetric color but random luminance (middle); or in nominally equiluminant images (bottom)**.

We also generated a further set of images to examine whether the chromatic contrast of the features influenced the face percepts. In this case the noise was created by randomly sampling the independent RGB values of each pixel, and then filtering the amplitude spectra to 1/f as before. The images were constructed to be symmetric in luminance while random in color or vice versa (Figure 8). A third set was constrained to be photometrically equiluminant. Nine new undergraduate observers were tested with this stimulus set. For these images the face percepts were very strongly dominated by the luminance contrast, such that observers again chose eyes based on regions of dark luminance levels (Figure 9). As a result, the feature locations were strongly symmetric when the luminance was symmetric, and asymmetric when the symmetry was instead confined to chromatic variations. For example, in Figure 8, a strong impression of symmetric faces is evident in the luminance symmetric noise, and is seemingly unaffected by the salient yet random variations in chromatic contrast. Conversely, this percept again breaks down when the symmetry is instead carried by the chromatic contrast. Nevertheless, when luminance was nominally removed (in the equiluminant images), the faces seen were again aligned to the axis of symmetry (Figure 9 right panel). This suggests that color itself could support some weak perception of faces. However, another possibility is that the choices were, instead, based on residual luminance variations in the images. Either way, the poor cue provided by color is consistent with deficits in face recognition as well as many other higher-order spatial judgments including shading and symmetry detection, when the information is carried by color rather than luminance (Gregory, 1977; Cavanagh, 1987; Morales and Pashler, 1999).

### Tests of face-like processing of faces from noise

**Figure 10:**
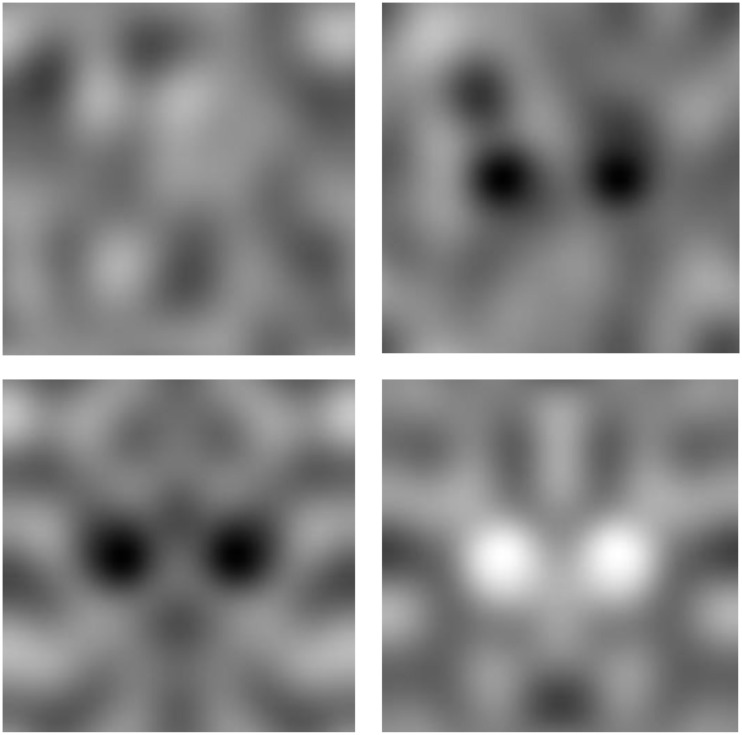
**Examples of images with “eyes” added to induce the percept of a face**. Top left: noise band-filtered to 2–4 cycles per image to reduce the number of potential features; top right: noise with dark spots added; bottom left: dark spots added to symmetric noise; bottom right: bright spots added to symmetric noise.

The preceding results show that symmetric filtered noise can lead to strong impressions of faces (at least when observers are explicitly instructed to search for faces in the noise). Moreover, these “faces” show many of the classic phenomena of face perception (e.g., inversion and negation as well as the importance of bilateral symmetry), suggesting that they may tap many of the configural processes thought to underlie face coding. The high density of highly articulated faces perceived further suggest that the stimulus configurations required to enlist these processes are fairly minimal and thus occur with high probability in the images. In fact, they may largely be triggered simply from a pair of dark spots. This led us in the second set of experiments to examine whether this minimal cue was sufficient to fully engage face processing. To assess this, we used a number of measures to compare visual performance for the noise faces and images of actual faces. Since the original noise images included multiple potential faces at many scales, we instead blurred the images by bandpass filtering them over a range from two to four cycles per image. We then added Gaussian blobs to the images to produce salient “eyes,” and asked how performance varied depending on the placement and polarity of the eyes and on the presence or absence of symmetry in the images (Figure 10).

#### Strength of face percepts

**Figure 11:**
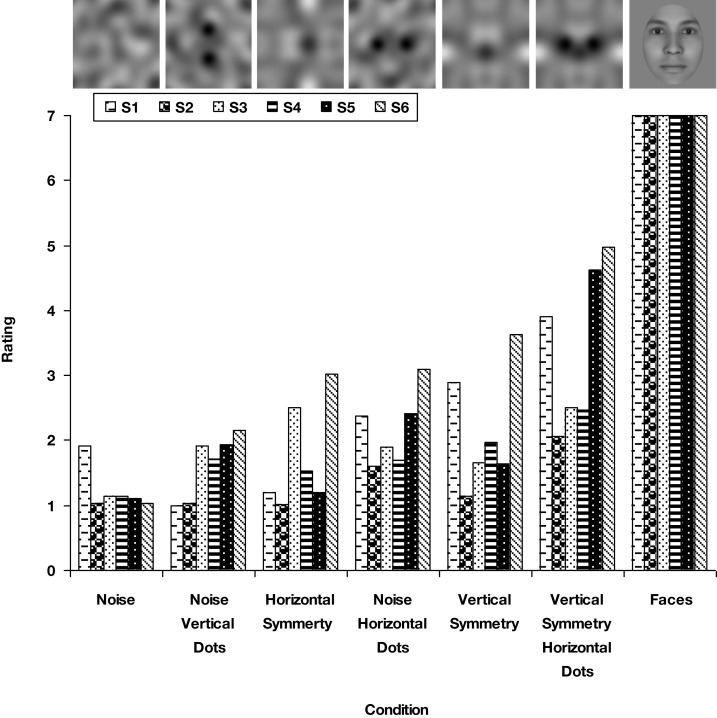
**Ratings of how “face-like” different versions of the noise appeared relative to a synthetic face image for six observers**. A value of seven indicated that the image strongly resembled a real face. Noise image categories are ranked from left to right in increasing average ratings.

As a preliminary test, we first measured how “face-like” the noise images appeared, by asking six observers to use a seven point scale to rate the degree to which the image resembled an actual face. Realistic synthetic face images (created with the program FaceGen) were, not surprisingly, uniformly scored as a fully articulated face (Figure 11). In contrast, the noise images were rated much lower. Nevertheless, they varied significantly in the strength of the face impression, being lowest for non-symmetric noise and highest for vertical symmetry with horizontal eye pairs. Thus the image structure again systematically varied the extent to which the impression of a face was evoked, so that we could next explore whether this impression was sufficient to modulate performance on other tasks.

#### Memory recognition for the noise images

In the first test, we asked whether noise samples would be easier to remember when they were perceived as a face, because the observer would form a stronger impression of the image’s “identity.” Faces are more easily remembered than non-face stimuli (Curby and Gauthier, 2007). We therefore asked whether noise that appeared more face-like could support better recognition. In this case the eyes were always added in the same place, and thus on their own carried no information for differentiating the images, and in fact reduced the information to the extent that they occluded parts of the background. Seven subjects were first presented a study set of images and then were tested with the same or new images in a recognition task. A wide range of pilot conditions (varying the number and duration of the stimuli) were assessed to vary the memory load and avoid floor and ceiling effects, yet in none of them did we find a recognition advantage for the more face-like images. Table 4 shows results for the final conditions chosen for formal testing. Participants were first exposed to a series of 10 study images shown for 3 s each, with each image defined by a different noise background but the same feature cue (noise only, noise plus horizontal dark spots, vertically symmetric noise with the dark spots, or vertically symmetric noise with bright spots). After a 0.5 s delay with white noise mask, a succession of 20 test images were displayed again for 3 s each. These included the 10 from the study set and 10 new images shown in random order, and for each the subject indicated whether the image was old or new. The task was repeated for the four different image blocks in counterbalanced order with the full sequence repeated five times for a total of 100 responses per image type. Accuracy under these conditions was generally low and averaged 60–70% across the different image sets. Although the noise was the least identifiable for nearly all observers, no consistent advantage was found for more face-like images, and no significant differences occurred across conditions as assessed by an analysis of variance [*F*(3,23) = 0.9, *p* = 0.46].

**Table 4:** **Recognition (new vs. old) rates for different classes of noise images in the memory task, based on the average responses of seven observers**.

	Mean correct (%)	Standard deviation
Noise	65.4	2.1
Noise + dark “eyes”	68.2	2.4
Symmetric noise + dark “eyes”	72.3	4.3
Symmetric noise bright “eyes”	69.7	3.0

The results suggest that the images which appeared more face-like were not, in fact, more easily remembered. It is possible that these images were not similar enough to a real face to facilitate recognition and memory. Another possibility is that even though images were face-like they appeared as very similar “individuals,” leading to an “other image” effect like the “other race” effect in which observers are poor at discriminating individuals from a population that differs from their own group. In any case, for these stimuli no recognition advantage was found for the noise images that were subjectively rated as more face-like in appearance.

#### Fixation patterns with the noise images

**Figure 12:**
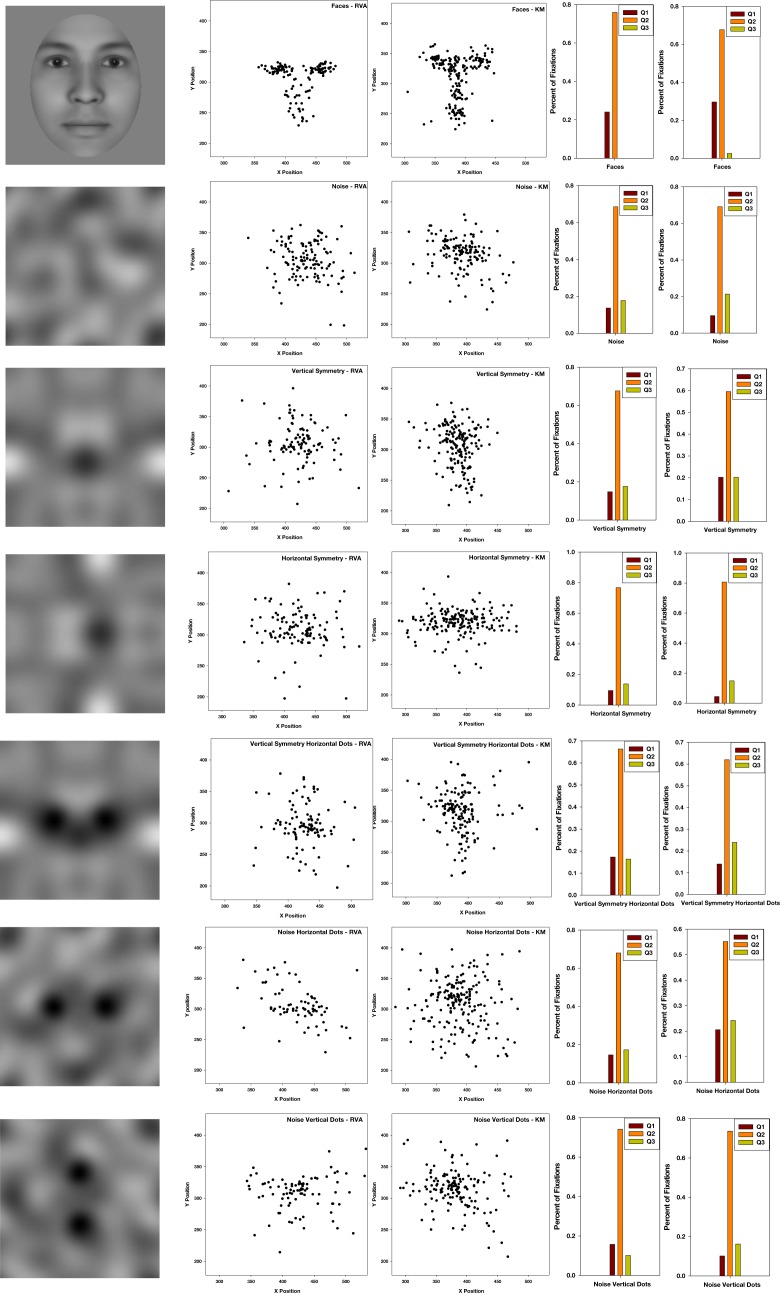
**Fixation patterns for two observers while scanning a realistic face or different versions of the noise images**. Histograms to the right show the number of fixations within a region containing the eyes or within regions above or below the eyes.

In the remaining tasks, we turned to responses that did not require distinguishing individuals but again simply processing the image as a face. In the first case we monitored eye movements to examine whether subjects would scan the noise images in different ways depending on whether or not they perceived them as faces. Observers tend to view actual faces with characteristic fixation patterns that focus on prominent facial features including the eyes and mouth (Yarbus, 1967). In fact plots of the fixation locations tend to recreate an image of the inspected face (Figure 12). If the added blobs induced a strong face percept, then observers might similarly focus their scans on the illusory eye and mouth/nose regions of the image. Four observers were tested and told to inspect a series of images while their eye movements were recorded with a CRS video eye tracker. They were instructed to scan each image to determine if it was the same as the image which preceded it. This was done to ensure the observers maintained attention and scrutinized each image. Twenty images from each category were displayed for 5 s each, with the category randomized across trials. Fixation points were defined as fixations lasting at least 60 ms with a distance of ten pixels away from the three previous averaged samples, which were recorded every 20 ms.

Figure 12 shows illustrative scan patterns for two observers. Again for the actual face the fixations were primarily around the eyes and nose and mouth. As a result observers spent more time looking below the eyes than above. We used this as a diagnostic for testing whether any of the noise sets might show a similar bias. Fixations were counted within a central band 60 pixels wide centered on the “eyes” (which were for the noise centered on the horizontal or vertical midline), as well as within quadrants defined by the remaining upper or lower sections of the display. A sign test was used to evaluate whether the proportion of fixations was greater in the region below the eyes than above. However, this failed to reach significance for any of the noise sets, and thus again did not reveal a face mode of processing for the noise images, even though some of these images produced a strong subjective impression of a face. Instead, a factor influencing the noise scanning was the axis of symmetry (along which observers tended to fixate more often), regardless of whether the eyes were present or absent.

#### N170 responses

In the final task we used event related potentials to test whether the noise faces could elicit an N170 response that is typically found for real faces (Bentin et al., 1996). Three participants were tested and were selected because they showed a high signal to noise ratio in their evoked potential responses based on a different set of prior experiments. Participants viewed eight categories of interleaved images. These included the seven categories used in the experiment rating the strength of face percepts (above), with the addition of a low contrast checkerboard which was added to serve as a baseline and completely non-face stimulus. Images were presented in categorical blocks where 60 different images within a category were shown serially for one second each, while observers passively viewed the sequence. Categories were presented rank ordered from least face-like, to most face-like to eliminate spurious effects due to priming (Bentin et al., 2002). Visual evoked potentials were recorded at the 10–20 system scalp location T6 with average ERP waveforms calculated for each stimulus category.

**Figure 13:**
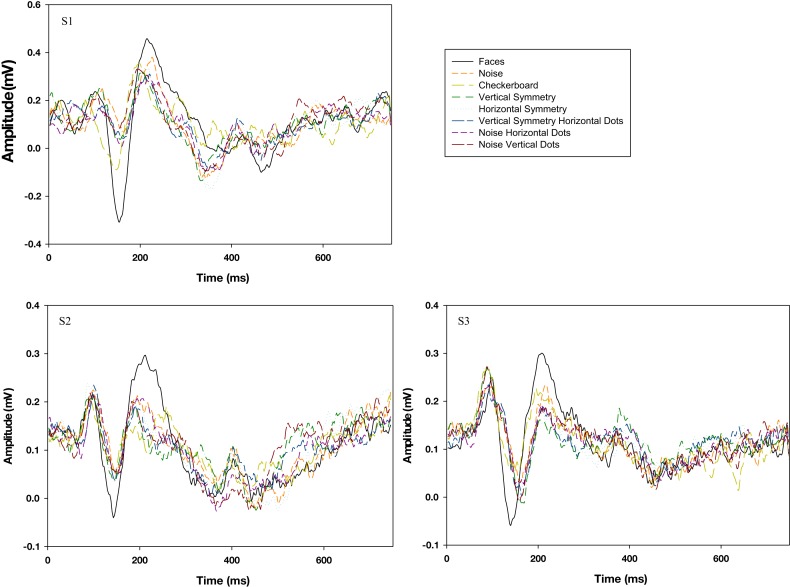
**Evoked potentials for face images or different variants of the noise images for three observers**. Responses to realistic faces are shown by the black trace and elicit the only clear N170 response.

Raw plots of the N170 amplitude versus latency for each observer are shown in Figure 13. Real faces elicited a strong N170 response. This was substantially larger than for any of the noise images, which in contrast showed little difference from each other. For example, normalized amplitudes of the N170 significantly differed for faces versus noise alone [t(2) = −15.6, p = 0.004] or versus the vertically symmetric noise with added eyes [t(2) = −21.6, p = 0.002]. In contrast, responses to these two noise patterns did not differ [t(2) = −2.25, p = 0.15], despite the fact that they varied substantially in their face-like appearance. The amplitude differences could potentially have occurred if the time to recognize the stimulus as a face were more variable for the noise (so that averaging across trials for the same stimulus onset washed out large peaks but with variable latency). However, this would predict longer average latencies for the noise images, which was not observed (e.g., mean latencies did not differ for the face and two noise types compared above [F(2,6) = 2.07, p = 0.21]. These results are limited to only a small sample of tested observers, and moreover cannot rule out the possibility that certain images within a given noise category might have evoked a stronger N170 (e.g., because they happened to resemble an actual face much more). However, like the preceding tasks, they are again consistent with the conclusion that the noise images fail to evoke some of the visual responses characteristic of actual faces.

## Discussion

In this study we examined the stimulus characteristics that give rise to face percepts, by asking observers to detect faces in images containing only noise. This approach thus follows in the tradition of classification images, in which perceptual templates are inferred by correlating how images are categorized with the parameters of the noise (Murray, 2011). Typically this involves adding noise to an actual stimulus in order to identify the image regions that are important for the percept. A number of studies have applied this approach to analyze the diagnostic image properties for face perception (e.g., Kontsevich and Tyler, 2004; Mangini and Beiderman, 2004; Sekuler et al., 2004; see also the related bubbles technique (Gosselin and Schyns, 2001; Smith et al., 2005). Previous studies have also explored face percepts in pure noise patterns. For example, Gosselin and Schyns (2003) assessed the perception of smiles in mouthless faces with added noise. Zhang et al. (2008) explored patterns of BOLD activation when observers looked for faces in pure noise, and recently extended this approach to examine behavioral responses to illusory faces (Rieth et al., 2011). They found that when an oval context for a head outline was provided, face percepts were triggered by simple configurations consistent with prominent internal (eyes, nose, mouth) but not external (e.g., ears) features, similar to the basic template we inferred. Without context the trigger features were more amorphous but still corresponded to dark regions, and notably, showed a bias for the left visual field.

In our study the image noise was again constrained to have a naturalistic amplitude spectrum (1/f) and to be symmetric about the vertical axis. As our images illustrate and results show, this had the advantage that the noise contained a remarkable profusion of perceived faces along the axis of symmetry. This allowed us to explore a large number of very well defined illusory faces in order to characterize the stimulus structure that gives rise to these illusions. Of course, this profusion might in part have resulted from priming the observers to specifically search for faces, and it is possible that the stimulus requirements for experiencing a face are more specific or qualitatively different when a face is instead perceived spontaneously. Nevertheless, the task we used measured whether “spontaneous” variations in the stimulus were consistent with a face-like percept. The stimulus characteristics that meet this criterion may thus provide a window into the early stages of human face perception where an image region is selected for processing as a face, and indicate some of the perceptual rules governing this selection.

What rules did our images reveal? The first, not surprisingly, was the importance of symmetry itself. Adding this property greatly facilitated the impressions of faces and completely dominated the salience of these percepts, as evidenced by the tight coupling of the chosen facial features to the midline. When the bilateral symmetry was removed observers could still find faces (e.g., Figures 7 and 9), but their prevalence was many times lower and the impressions were much less distinct. (It is possible that in addition to symmetry there was a bias to look for faces along the vertical midline or “middle” of the image. However, Figures 7 and 9 suggest this bias must be a much weaker factor, and moreover as noted above, Rieth et al. (2011) instead found a left field bias in asymmetric noise). Symmetry is a highly conspicuous feature that produces strong activity in higher visual areas (Sasaki et al., 2005), and is likely to be an important configural dimension in face coding (Rhodes et al., 2005). For example, attractiveness depends in part on facial symmetry (Rhodes et al., 1998; Perrett et al., 1999). Moreover, when observers are exposed to asymmetric faces they adapt so that the faces tend to appear more symmetric, consistent with a normalization for this configural dimension (Paras et al., 2004; Morikawa, 2005; Rhodes et al., 2009).

A second prominent and predictable property was the importance of orientation. Virtually all of the faces reported were upright, and completely different faces were perceived when the same images were inverted. In this regard our stimuli resemble classic “double-face” illusions in which a face is depicted so that a different upright face can be seen in either orientation. Analogous to these, the failure to detect inverted faces in our images likely reflects the dense spatial structure of the noise, so that competing upright faces were always present in the same locations. The higher salience for upright faces thus masked the perception of faces at other orientations (Martini et al., 2006). In informal observations, we also found that observers were much less likely to perceive faces when the images were instead symmetric about the horizontal axis.

A third property was that eyes were the singularly critical feature for detecting a face. These were reported in nearly all faces, while all other features appeared optional and thus subordinate. The importance of the eye region for face perception is well documented (Itier and Batty, 2009). In fact the powerful influence of symmetry may in part be because it increases the prevalence of corresponding eye pairs. Notably, there were strong constraints on which pairs were seen as eyes. In particular, asymmetric eyes were rarely reported within symmetric images, and eye size closely scaled with eye separation (e.g., so that small spots were very unlikely to be reported as eyes if they were far from the midline). This could be because in these cases there is a high probability of competing structure in between. However, it suggests that the perceptual template for eyes is not simply a pair of dots but rather a triplet of features (two eyes with ∼nothing in between) and that the ratio of size to spacing is strongly constrained.

The observers’ percepts confirmed another likely fundamental property of face coding that it is strongly polarity selective. Again, the regions identified as eyes were strongly biased toward darker spots, while features such as the nose or mouth could be dark or bright. Moreover, contrast polarity completely overrode the cue of spatial symmetry. The impact of polarity is consistent with the dramatic disruption of face recognition with contrast negation (Russell et al., 2006), and with the special role that eye polarity plays in this effect. For example, photographic negatives remain recognizable when the region of the eyes and brow are not inverted (Gilad et al., 2009). This may also explain why faces take on an especially unnatural appearance when the eyes are depicted as a uniform white rather than dark area.

Finally, our results also suggest that the processes underlying these percepts are largely color blind. As illustrated in Figure 8, strong face impressions remained when randomly varying color was added to the luminance symmetric images, while color symmetry failed to elicit clear faces in the presence of random luminance. Again, this is not unexpected, for color variations do not support the perception of symmetry (Morales and Pashler, 1999), which appeared to be a dominant cue for seeing faces in our images. Moreover, color provides a poor cue to shading (Cavanagh, 1987), and notably a recent study found that this deficit more strongly impact the recognition of faces than objects (Pearce and Arnold, 2012). Color has nevertheless been shown to support some facial judgments such as gender (Nestor and Tarr, 2008), and may be fundamentally important in judging attributes of faces from their complexion, such as their expression or health (Changizi et al., 2006). However, our results suggest that the processes mediating these judgments are largely independent from those supporting facial percepts based on spatial information (Webster and MacLeod, 2011). Consistent with this independence, studies of adaptation have failed to reveal that color aftereffects can be made contingent on identifiably different facial configurations (Yamashita et al., 2005).

As noted, one aim of our study was to examine the extent to which detecting a stimulus as a face engaged configural or holistic processing. This is not *a priori* necessary because face perception might depend on both holistic and analytical representations (Moscovitch et al., 1997), and our stimuli were chosen to assess the minimal requirements for detection. Moreover, what constitutes evidence for face-specific or for configural processing remains debated (Sekuler et al., 2004; Martelli et al., 2005; McKone et al., 2007). However, two aspects of the results are at least consistent with configural coding. First, as noted above, the selected faces demonstrated many of the assumed canonical properties of configural coding, including the importance of symmetry and orientation. Second, as we have also emphasized, the faces seen often appeared highly individuated and articulated, and thus appeared to support the fine within-class discriminations that are thought to depend on configural coding. Such results are compatible with other recent evidence suggesting that configural and holistic processing are in fact inextricably linked with the initial stages of face detection (Richler et al., 2009; Taubert et al., 2011).

But why were so many of the faces seen in these noise images so clear and distinct? One part of this answer is that the 1/f noise we used provides a rich spatial structure with features and potential configurations visible at many scales (Field and Brady, 1997). Further, our results suggest that the stimulus requirements for eliciting a face percept are very minimal and thus very general, so that they have a high probability of arising by chance. In particular, a pair of dark spots was usually adequate, and these pairs are very common in the symmetric images we used. They also arise with high frequency in many natural images, such as clouds and rocks, which similarly have roughly fractal structure, and this can account for the high tendency for observers to report faces in natural textures and scenes. However, the presence of these basic trigger features leaves the question of why the faces so often appear vivid. As noted, it is likely that once an image is coded as a face, the remaining features and variations in the image are re-interpreted to be consistent with this representation. Thus random lines become cheekbones or brows or wrinkles, and the image suddenly takes on a highly specific and detailed rendering as a particular face. Thus the percepts appear to strongly reflect a face mode of processing in which top-down inferences shape and perceptually complete the interpretation. This itself represents a strongly holistic process, for how one noise feature is interpreted (e.g., as an eye) can completely change the perception of other nearby features (Tanaka and Farah, 1993). These two processes also explain the seeming paradox that faces can be seen almost anywhere (because the trigger features are minimal), yet the faces that are seen are surprisingly distinctive and thus appear exceptionally improbable (because this depends on the full integration of the random pattern). It is intriguing that the general public is aware of the seeming impossibility of seeing an individual’s likeness in a random pattern, such that a face seen in toast continues make headlines, and that their captivation arises because they attribute the apparition to the object rather than the observer.

Given that a pair of dark spots can evoke such a rich experience of a face, we were led in the second set of experiments to test whether they also evoked other characteristics of face processing. However, in the three cases we tested – recognition memory, scanning patterns, and event related potentials – the “eyes” did not have it. This may in part be because we chose to use highly impoverished stimuli in order to provide a minimal configuration for the task, for in the 1/f noise images the impressions often came close to resembling actual faces. However, these sparse stimuli did appear face-like to observers, yet did not support a face-specific response like the N170, even though a strong response can be elicited by eyes alone (Bentin et al., 1996). This dissociation is similar to a recent analysis of cortical responses to face-like images, sampled from false alarms generated by a face recognition algorithm (Meng et al., 2012). BOLD responses in the right hemisphere showed a categorical influence on whether or not the image was perceived as a face, while responses in the left hemisphere were instead graded with facial resemblance. Such results suggest that the multiple processing stages involved in the visual analysis of faces may have very different thresholds or stimulus requirements. Here we have shown that a highly restricted and simple set of image properties can support strong and vivid percepts of facial configurations even if they fail to fully engage all mechanisms of face coding.

## Conflict of Interest Statement

The authors declare that the research was conducted in the absence of any commercial or financial relationships that could be construed as a potential conflict of interest.
